# Content Analysis of Metaphors About Hypertension and Diabetes on Twitter: Exploratory Mixed-Methods Study

**DOI:** 10.2196/11177

**Published:** 2018-12-21

**Authors:** Lauren Sinnenberg, Christina Mancheno, Frances K Barg, David A Asch, Christy Lee Rivard, Emma Horst-Martz, Alison Buttenheim, Lyle Ungar, Raina Merchant

**Affiliations:** 1 Penn Medicine Center for Digital Health University of Pennsylvania Philadelphia, PA United States; 2 Department of Family Medicine and Community Health Perelman School of Medicine University of Pennsylvania Philadelphia, PA United States; 3 Center for Health Equity Research and Promotion Philadelphia Veterans Affairs Medical Center Philadelphia, PA United States; 4 Computer and Information Science University of Pennsylvania Philadelphia, PA United States; 5 Department of Emergency Medicine Perelman School of Medicine University of Pennsylvania Philadelphia, PA United States

**Keywords:** cardiovascular diseases, language, metaphor, social media, hypertension, diabetes mellitus

## Abstract

**Background:**

Widespread metaphors contribute to the public’s understanding of health. Prior work has characterized the metaphors used to describe cancer and AIDS. Less is known about the metaphors characterizing cardiovascular disease.

**Objective:**

The objective of our study was to characterize the metaphors that Twitter users employ in discussing hypertension and diabetes.

**Methods:**

We filtered approximately 10 billion tweets for keywords related to diabetes and hypertension. We coded a random subset of 5000 tweets for the presence of metaphor and the type of metaphor employed.

**Results:**

Among the 5000 tweets, we identified 797 (15.9%) about hypertension or diabetes that employed metaphors. When discussing the development of heart disease, Twitter users described the disease as a journey (n=202), as transmittable (n=116), as an object (n=49), or as being person-like (n=15). In discussing the experience of these diseases, some Twitter users employed war metaphors (n=101). Other users described the challenge to control their disease (n=34), the disease as an agent (n=58), or their bodies as machines (n=205).

**Conclusions:**

Metaphors are used frequently by Twitter users in their discussion of hypertension and diabetes. These metaphors can help to guide communication between patients and providers to improve public health.

## Introduction

### Communicating About Health Through Metaphors

Humans often communicate about health using metaphors. Physicians were found to use metaphors in 64% of conversations with patients with serious illness [1], and patients also use metaphors when discussing their own illnesses [2].

Prior work has focused on characterizing metaphors used to describe patients’ experience of cancer, tuberculosis, and HIV [3-5]. Although cardiovascular disease affects more than 80 million Americans, less is known about the metaphors used by patients to describe heart disease [6]. Characterizing these metaphors might inform patient-provider communication by promoting understanding of the public’s framing of disease.

Metaphor is an efficient way to link conceptual domains, which allows us to make inferences among seemingly unrelated items or events [7]. Taken in the aggregate, metaphors can help us understand higher-order cultural frameworks about ways that the world works. Finally, metaphors help to perform the task of analogic reasoning [8].

### Objective

Prior work has evaluated health-related metaphors largely through surveys and patient recall. These approaches may miss the metaphors of day-to-day conversation between people. The social media network Twitter is increasingly used by researchers to analyze patient language around health [9]. Previously, we showed that almost half of a sample of cardiovascular-related tweets contained metaphorical language [10]. In this study, we expanded this work and characterized metaphors in tweets about hypertension and diabetes.

## Methods

### Study Design

This was a sequential, exploratory, mixed-methods study of Twitter data relating to hypertension and diabetes.

### Data Source

#### Identification of Tweets

To identify tweets about hypertension and diabetes, we used the standard Twitter application programming interface (Twitter, Inc, San Francisco, CA, USA) [10].

From a random 10% sample of tweets over the 6-year period from July 2009 to February 2015, we searched approximately 10 billion tweets for keywords related to hypertension and diabetes [11,12]. To generate these keywords, we used the Unified Medical Language System, Consumer Health Vocabulary, and the agreement of study authors. Keywords identified were hypertension (high blood pressure) and diabetes (blood sugar, mellitus) [10-12].

We limited our analysis to English-language tweets that could be mapped to a US county. Additional details about Twitter processing and data extraction are described elsewhere [10].

#### Metaphor Coding

Metaphor was defined as a representation of a subject rather than the subject itself, or a non–reality-based statement with an analog in the real world.

To identify tweets containing metaphors, 2 coders (CLR and CM) evaluated a random subset of 1000 tweets about hypertension and 4000 tweets about diabetes for the presence of metaphor, adjudicating differences with a larger group that included 3 other authors. The characteristics of the metaphors were coded into metaphor types. These 5000 tweets represented approximately 1% of the total tweets returned using the search strategy above. After coding 5000 tweets, total agreement in each category was greater than 90% with a kappa score of .77.

We performed data coding and analysis in NVivo version 10.0 (QSR International).

## Results

Of the 4000 diabetes tweets, 12.1% (n=482) contained metaphors, while 31.5% (n=315) of the 1000 hypertension tweets contained metaphor. We further categorized metaphorical tweets into 2 groups: metaphors that described the development of disease and those that described the experience of disease.

### Development of Disease

Four themes in the development of disease emerged: journey to disease, disease as transmittable, disease as object, and disease as actor (Table 1).

#### Journey to Disease

Many tweets described acquisition of the disease as a journey that originated with a “vector” and travelled along a path to the person (202/312, 64.7%). These tweets described the disease as being acquired through a single encounter (eg, “You can probably get high blood pressure from one large fry at McDonald’s”) or described developing the disease as a culmination of many influences (eg, the hashtag *#* roadtodiabetes).

#### Disease as Transmittable

Other Twitter users described hypertension and diabetes as transmittable diseases (eg, “Don’t talk to me, you’re giving me diabetes” or “I have to avoid diabetes like the plague”).

#### Disease as Object

Some users described the disease as an object that was tangible. Often these tweets contained metaphors about the disease existing within food (eg, “I could taste the diabetes”). This metaphor was more frequently used in the context of diabetes than in hypertension (48/211, 22.7% vs 1/101, 1.0%).

#### Disease as an Actor

Some tweets labeled diabetes and hypertension as sinister agents (eg, “Hypertension dangers lurking in obese kids”).

### Experience of Disease

Four themes in the experience of disease emerged: body as machine, disease as difficult to control, war and destruction, and disease as agent (Table 1).

#### Body as Machine

Many tweets represented the body as a machine and the disease as an interruption of the machine’s functioning (eg, “Shuttling blood sugar into cells”). These mechanical metaphors were more frequently used to describe hypertension (167/214, 78.0%) than diabetes (38/271, 14.0%).

#### Disease as Difficult to Control

Some tweets used metaphors that helped to demonstrate the difficulty of controlling one’s disease (eg, “Stepped care approach to tackle uncontrolled high blood pressure”).

**Table 1:** Metaphors used in tweets to describe the development and experience of diabetes and hypertension^a^.

Metaphor themes		Diabetes, n (%)	Hypertension, n (%)	Example tweets
**Development of disease (diabetes: n=211; hypertension: n=101)**				
	Journey to disease	140 (66.4)	62 (61.4)	You can probably get high blood pressure from one large fry at McDonald’s #roadtodiabetes Tips on how to prevent the path to diabetes Couples who develop diabetes together stay together! I really feel the diabetes coming
	Transmittable	53 (25.1)	63 (62.4)	I’d rather catch diabetes than feels I have to avoid diabetes like the plague Stop! you’ll give me diabetes... Don’t talk to me, you’re giving me diabetes. Salty enough to give high blood pressure
	Disease as object	48 (22.7)	1 (1.0)	I could taste the diabetes Mountain dew tastes like diabetes and depression Diabetes for breakfast tomorrow yay I asked for a brownie and instead I got diabetes That’s a nice cart full of diabetes you’ve got there
	Disease as actor	12 (5.7)	3 (3.0)	Hypertension dangers lurking in obese kids When high blood pressure attack No diabetes allowed Like sugar? So does...diabetes Don’t die on me! No diabetes allowed. LOL
**Experience of disease (diabetes: n=271; hypertension: n=214)**				
	Mechanical	38 (14.0)	167 (78.0)	Actually, beer IS good for lowering high blood pressure Shuttling blood sugar into the cells My blood sugar drops drastically today...I feel like my energy has been drained. Still feeling weak until now. I be low then I be high it’s like a battle knife to a gun fight no need to worry it’s the story of my life blood sugar I just had a crazy blood sugar spike
	Control	31 (11.4)	13 (6.1)	Control your high blood pressure Stepped care approach to tackle uncontrolled high blood pressure The best exercise to control high blood pressure seems to be virtually any exercise....Get up and get going! Telehealth may help patients control high blood pressure, but engagement is a barrier The message is to prevent and control diabetes
	War	84 (31.0)	17 (8.0)	Diabetes destroyer In the fight against high blood pressure, potassium is a powerful weapon Simple Tips On How to Battle Diabetes The American Diabetes Association is leading the fight against the deadly consequences of diabetes Slash high blood pressure
	Disease as actor	38 (14.0)	20 (9.3)	Hypertension is a silent killer Oh diabetes, you’re the reason I don’t sleep Diabetes messing with my eyes Diabetes doesn’t define me, it explains me My blood sugar plays funny little tricks on me

^a^Percentages equal more than 100%, as some tweets fit into more than one theme.

#### War and Destruction

Some tweets used militaristic metaphors, including words such as “destroyer,” “fight,” and “battle” (eg, “The American Diabetes Association is leading the fight against the deadly consequences of diabetes”).

#### Disease as an Actor

As in the tweets describing the development of disease, in tweets about the experience of disease, users often represented diabetes or hypertension as agents of evil (eg, “Hypertension is a silent killer. Monitor your BP [blood pressure] now...”).

## Discussion

### Principal Findings

This study had two main findings. First, metaphor is a substantial part of how Twitter users communicate information about hypertension and diabetes. Second, the conceptualization of disease implied by the metaphors described often conflicts with biomedical understanding of the biological underpinnings of disease.

In the 1970s, Susan Sontag described the widespread use of metaphors in communication about cancer and HIV/AIDS [5]. She argued that the use of metaphorical language about illness could adversely influence public attitudes or clinicians’ practices and, in the end, patients’ experience.

In contrast, less has been studied about heart disease. A better understanding of metaphors about hypertension and diabetes offers promise for understanding what a population thinks about the etiology, treatment, and progression of disease. This “code” could provide insights into a disconnect between a health condition and its treatment and management.

Metaphors may be used by patients seeking to discuss their condition with family and friends and to relate to other patients experiencing the same disease. Patients affected by diseases associated with great morbidity and mortality may have greater motivation to formulate conceptual frameworks to explain their disease, allowing them to better cope with the complex, medical reality of their condition.

According to our results, the general population’s understanding of hypertension seems to be narrower than that of diabetes. A higher percentage of metaphors were employed in the sample of hypertension tweets than in the diabetes tweets, yet the majority of those metaphors associated hypertension with a purely mechanical understanding of the experience of the disease, such as the raising or lowering of pressure within the body (167/214, 78.0%). Patients may have fewer approaches to thinking about hypertension because it is less salient than diabetes—that is, it is less likely to cause direct symptoms, less likely to require time-intensive management on the part of the patient, and less likely to be perceived as fatal.

While some of the metaphor themes we found have been previously described in the literature, others are newly described as metaphor, such as disease as object or disease as actor [5,13]. These metaphors may be new because of our focus on heart disease and diabetes rather than cancer or infectious disease, or perhaps because Twitter provides a window into the public’s spontaneous language about disease.

While some of the metaphors described here are consistent with our biomedical understanding of disease (eg, body as machine), other metaphors are incongruent with the pathophysiology of the disease (eg, disease as object, disease as transmittable). Many of these metaphors were employed in jest. It remains unclear whether the use of metaphors inconsistent with the medical community’s understanding of disease represents a misunderstanding to be corrected, or an effective rhetorical approach to be appropriated.

### Limitations

This study had several limitations. This study characterized only US-based English-language tweets; additional metaphors would have emerged if we had studied a broader sample of tweets. While we removed duplicate tweets, we counted each retweet individually in this study, as we believed that the propagation of any given message reflects the salience of that metaphor.

### Conclusions

Metaphors are windows into the shared understandings that laypeople have about illness. They have potential to provide physicians with tools to explain difficult scientific concepts in a culturally appropriate context. However, these benefits must be balanced with the potential harm that metaphors can induce by propagating inaccurate or potentially stigmatizing language [3].
